# Seroprevalence of Human Enterovirus 71 and Coxsackievirus A16 in Guangdong, China, in Pre- and Post-2010 HFMD Epidemic Period

**DOI:** 10.1371/journal.pone.0080515

**Published:** 2013-12-04

**Authors:** Wei Li, Lina Yi, Juan Su, Jing Lu, Changwen Ke, Hanri Zeng, Dawei Guan, Cong Ma, Wanly Zhang, Hong Xiao, Hui Li, Jinyan Lin, Yonghui Zhang

**Affiliations:** 1 Center of Pathogen Detection Research for Emerging Infectious Diseases, Guangdong Provincial Center for Disease Control and Prevention, Guangzhou, China; 2 Key Laboratory of Pathogen Detection for Emergency Response, Guangdong Provincial Center for Disease Control and Prevention, Guangzhou, China; 3 Key Laboratory of Depository and Application for Pathogenic Microbiology, Guangdong Provincial Centre for Disease Control and Prevention, Guangzhou, China; 4 Guangdong Provincial Institute of Public Health, Guangdong Provincial Center for Disease Control and Prevention, Guangzhou, China; Public Health England, United Kingdom

## Abstract

**Background:**

Human Enterovirus 71 and Coxsackie A16 have caused many outbreaks in the last decade in mainland China, resulting in thousands of fatal cases. Seroepidemiology which provides important information to document population immunity is rare in China.

**Methodology/Principal Findings:**

A cross sectional study of Enterovirus 71 (EV71) and Coxsackie A16 (CA16) seroprevalence was carried out in Guangdong, China, pre- and post- the 2010 hand, foot and mouth disease (HFMD) epidemic period. The levels of EV71 and CA16 specific antibodies were evaluated by a microneutralization test and the geometric mean titer (GMT) was calculated and compared. Our results indicated frequent infection by EV71 and CA16 in Guangdong before the 2010 epidemic. Only EV71 neutralizing antibody but not CA16 seroprevalence was significantly increased after the 2010 HFMD epidemic. Children less than 3 years old especially those aged 2 years showed the lowest positive rates for EV71 and CA16 NA before epidemic and the most significantly increased EV71 seroprevalence after epidemic. CA16 GMT values declined after the 2010 epidemic.

**Conclusions:**

These results indicate EV71 was the major pathogen of HFMD in Guangdong during the 2010 epidemic. The infection occurs largely in children less than 3 years, who should have first priority to receive an EV71 vaccine.

## Introduction

Hand, foot and mouth disease (HFMD) is a common and highly infectious disease [1]. Characterized by fever, mouth ulcers and rash on hands and feet, the HFMD as itself is usually mild and self-limited [2], [3]. Occasionally, when accompanied with neurological complications (e.g., encephalitis), severe organ impairment and even death can occur [4], [5]. Belonging to the picornaviruses, Enterovirus 71(EV71) and Coxsackie A16 (CA16) are commonly recognized as the cause of HFMD [6], [7]. Compared to CA16, EV71 is more often associated with severe HFMD cases [8]. There are no specific therapies to treat severe HFMD cases [9]. EV71 vaccine candidates are being developed [10], [11], [12]. Recently, an effective vaccine for EV71 at phase 3 trial has been reported in China [13].

Several large outbreaks of HFMD associated with severe and fatal outcomes have been reported in the last decade in Southeast Asia, especially in mainland China [6], [14], [15], [16], [17]. Sporadic HFMD cases have been identified in most provinces in mainland China since the first report in Shanghai in 1981 [18]. However, HFMD was not notifiable to the Ministry of Health in China until 2008 when it was classified as a category C Notifiable Infectious Disease after a big outbreak of HFMD in Fuyang, China, in April 2008. Thereafter, an unprecedented HFMD epidemic occurred in 2010 in mainland China [15], [16]. According to the surveillance data in national Center for Disease Control and Prevention, a total of 3,419,149 HFMD cases including 1384 deaths were reported by the end of 2010 [19]. In Guangdong province, 230,978 children were diagnosed with HFMD, resulting in an incidence rate of 235 per 100,000 [20]. EV71 was diagnosed in nearly all of the severe cases in 2008 [20], [21]. Surveillance data collected by Center for Disease Control and Prevention of Guangdong (GDCDC) indicates same situation in 2010 (unpublished data).

Seroepidemiology provides information of great importance to assure population immunity [22]. Herd immunity of certain viruses increased after outbreaks [23]. However, only a few of serum surveys have been conducted on EV71 and CA16 in China [19], [24], [25]. The scale of EV71 and CA16 viral transmission in healthy individuals after an outbreak of HFMD was largely unknown. In this study, the seroprevalence of EV71 and CA16 before and shortly after the 2010 epidemic in Guangdong province was investigated and the changes both in exposure rates and immunity levels were analyzed.

## Materials and Methods

### Human Subjects and Serum Samples

The material used in this study is stored serum samples collected from individuals who had participated in a previous influenza virus study at Guangdong Provincial Centre for Disease Control and Prevention, China, 2010 [26]. Briefly, a multi-stage stratified and cluster random sampling was applied for sample selection according to the residency address. By using a random digits table, five urban districts from the capital-city and twenty districts/counties from twenty middle and small-sized cities were selected and remained unchanged in the two time periods. The investigating team obtained a name list of all individuals (including age) residing in the street/town and randomly selected individuals from the name list. No same individuals contributed to the pre- and post- sampling. Survey questionnaire was completed by trained interviewers and included information on the subject’s age, gender, vaccination history (over the past year) and presence/absence of illnesses (over the past year). The serum samples were stored at −80°C until testing. Our previous epidemiology studies indicated the majority of EV71 infection occurred in preschool-aged children with the HFMD epidemic peak appeared in May and June [21]. To study the seroprevalence before and after the major outbreak in 2010, we chose serum samples from January 8 to January 24 and from August 23 to September 4 respectively. Participants were included if they showed no sign of disease at the time of sample collection. Serum samples were classified into seven age groups (1, 2, 3, 4, 5–20, and ≥21 years). Each group has 47–81 samples, except age group≥21 years before epidemic(38 samples) and 3, 5–20 years group after epidemic(41 and 98 samples respectively). The overall sex ratio of boys to girls was 1.2∶1. The demographic profile of the participants enrolled in this study was illustrated in Table 1. For the use of serum samples, written informed consent from all participants (or their parents/legal guardians) involved in the study were obtained. The study was approved by the ethics committee of the Guangdong Provincial Center for Disease Control and Prevention, and was in compliance with the Helsinki Declaration.

**Table 1:** Demographic profile of the subjects.

	No. samples in each age group (years)					
	1	2	3	4	5–20	≥21
**Before**						
Male	33	38	40	42	29	20
Female	27	27	34	39	21	18
Total	60	65	74	81	50	38
**After**						
Male	24	32	21	33	54	23
Female	25	16	20	23	44	24
Total	49	48	41	56	98	47

No: number; before: before the 2010 HFMD epidemic; after: after the 2010 HFMD epidemic.

### Neutralizing Antibody Assay

All of the neutralizing antibody (NA) assays were run in 96-well micro plates and performed as previously described [27]. EV71/Guangdong/EV039/2009 (C4 genotype) and CA16/Guangdong/CA010/2009 (A genotype) strain was used to quantify neutralizing antibody levels for EV71 and CA16 respectively. Serum samples were inactivated at 56°C for 30 minutes before use, diluted two-fold from 1∶8 to 1∶1,024, and then incubated at 37°C for 2 hours with equal volumes of 100 half tissue culture infective doses (100 TCID50) of EV71 or CA16. After the incubation period, 1×10^5^ cells/ml rhabdomyosarcoma cell lines (RD) were added to each well. Finally, these plates were incubated in a 5% CO2 incubator at 37°C for 7 days. Cytopathic effect (CPE) was observed with an inverted microscope from the fourth day. All the diluted samples were tested in duplicate. Cell control, serum control and virus control were included in each plate. Viral back titration was conducted in each test. The antibody titer of the sample was defined as the highest dilution that could inhibit CPE development in 50% of the virus-infected wells. A titer equal to or greater than 8 was considered as seropositive [19].

### Statistical Analysis

Statistical analyses were performed with SPSS version 13.0 (SPSS Inc., Chicago, IL, USA). We calculated the proportion of seropositive participants for each age group, time period (before and after epidemic) and sex with 95% confidence intervals according to the binomial distribution. To compare the overall seroprevalence before epidemic with that of after epidemic, the univariate logistic regression model and multivariate regression model (with sex and age being controlled) were performed. Crude odds ratio (OR, seroprevalence after vs. before epidemic) and adjusted odds ratio with 95% confidence intervals (CI 95%) were calculated. The chi-square test was used to compare the differences in the seroprevalence among different groups between two time periods (overall seroprevalence before vs. after epidemic; male seroprevalence before vs. after epidemic; female seroprevalence before vs. after epidemic; seroprevalence in each age group before epidemic vs. after epidemic). To compare the seroprevalence among different age groups in each time period, we re-classified age group into two levels (age< = 3 and age>3). A logistic regression model with sex being controlled was performed. Statistical significance was established assuming an alpha error of 0.05. The geometric mean titer (GMT) and corresponding 95% CI were computed as describe previously [28]. Briefly, we first took the logarithmic transformation (base 10) of the titer readings, followed by antilog transformation of the mean and its 95% CI [29]. To compare the overall GMT value before epidemic with that of after epidemic, the linear model with time (age and gender were controlled) was performed. GMT comparison between two time periods (before vs. after) in each age group was performed by using Kruskal–Wallis test [19]. Antibody titers >1∶1204 were assigned a value of 1204. Antibody titers <1∶8 were assigned a value of 4 [25], [30].

## Results

### Seroprevalence of EV71 or CA16

**Figure 1:**
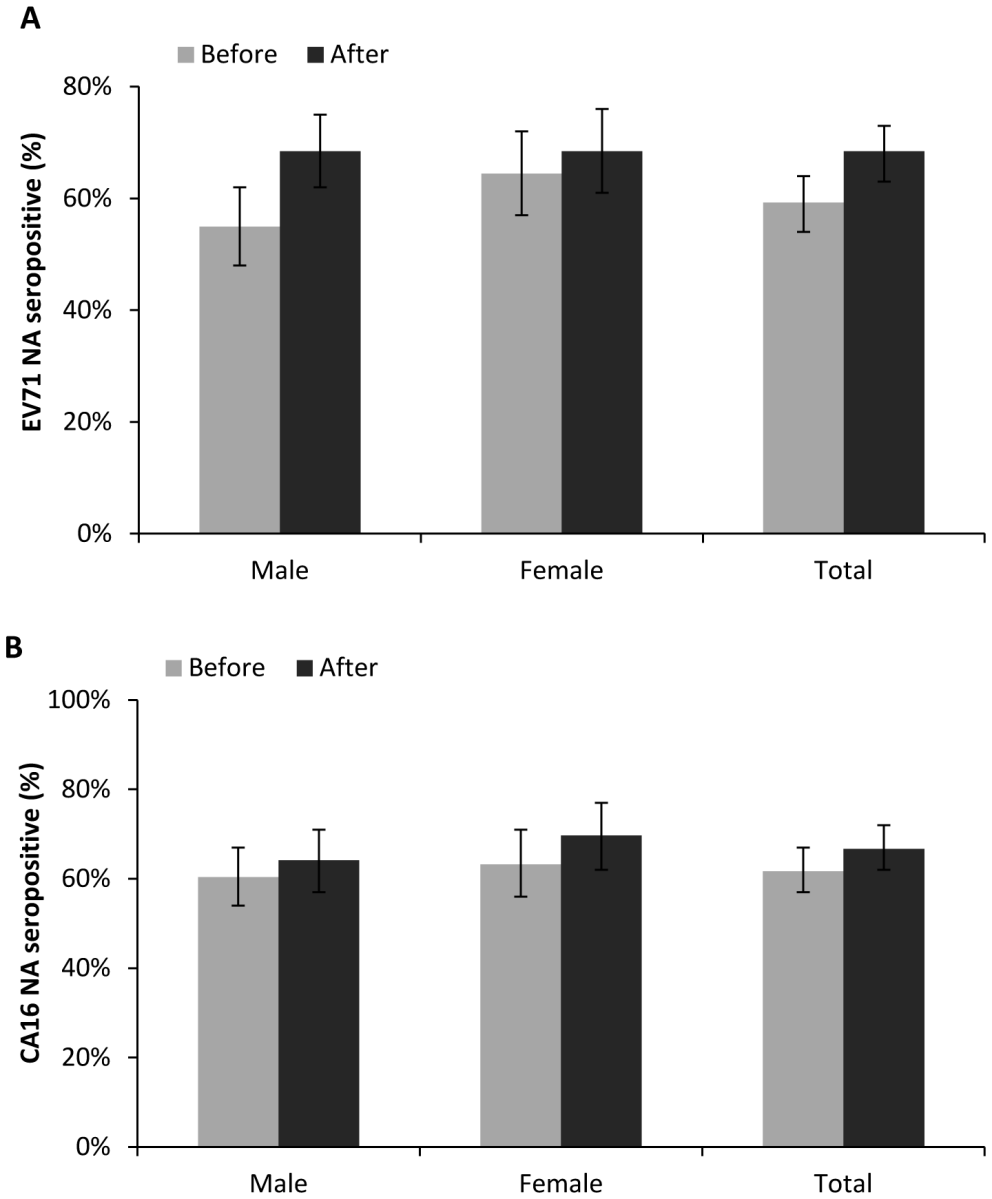
Overall seroprevalence of EV71 and CA16 antibody. Overall seroprevalence of neutralizing antibodies to EV71 (A) and CA16 (B) in individuals in Guangdong, China, before and after the 2010 HFMD epidemic. Before: before the 2010 HFMD epidemic; after: after the 2010 HFMD epidemic. The lines indicate 95% confidence interval.

Serum samples from healthy individuals were obtained and tested for EV71 and CA16 neutralizing antibody (NA). Before the large 2010 HFMD epidemic, similar proportion of participants identified seropevalence for EV71 and CA16 NA, with positive rates of 59.2% (95% CI 54.2–64.1%) and 61.7% (95% CI 57.4–67.6%) respectively (Fig. 1). The seroprevalence for EV71 increased significantly after the 2010 HFMD outbreak (crude OR = 1.49, p = 0.01; adjusted OR = 1.99, p = 0.02). Among the 339 tested individuals, 232 (68.4%, 95% CI 63.4–73.6%) had detectable EV71 antibody titers (Fig. 1A). No significant changes were identified for CA16 serostatus(Fig. 1B). The seropevalence was 66.7% (95% CI 62.4–72.6%) after the outbreak (crude OR = 1.24, p = 0.17; adjusted OR = 1.20, p = 0.25).

The EV71 seroprevalence before epidemic were 55.0% and 64.4% for male and female respectively, (Fig. 1). Then values increased both to 68.4% after the epidemic, with significantly increased male seroprevalence observed after the epidemic (P<0.01). While for CA16, 60.4% and 64.2% of males were tested NA positive before and after, compared to 63.2% and 69.7% among females, with no significant gender specific difference observed.

### Age-dependent Seroprevalence of EV71 or CA16

**Figure 2:**
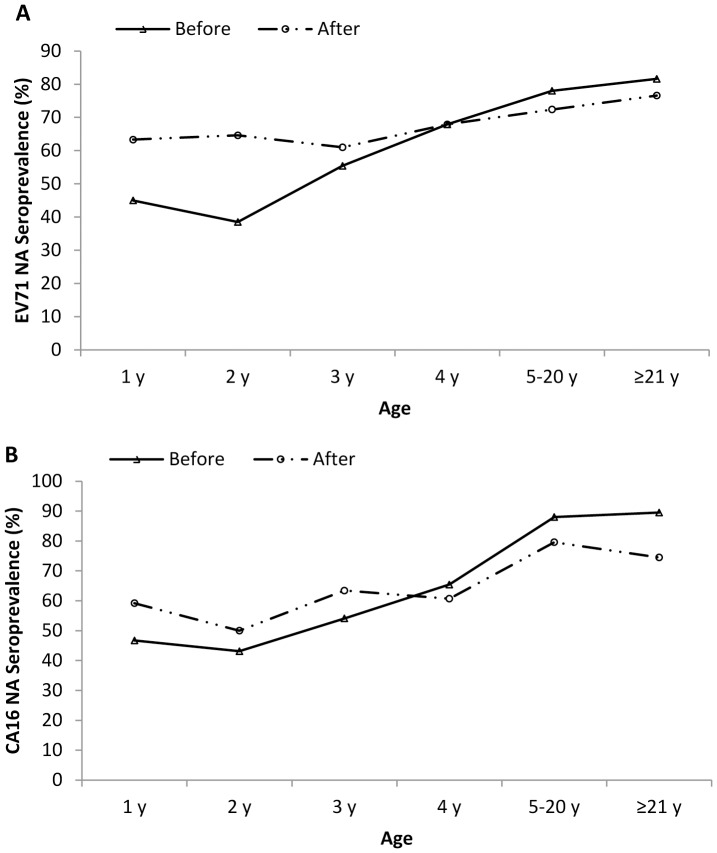
Age-dependent seroprevalence of EV71 and CA16 antibody. Age-related seroprevalence of neutralizing antibodies to EV71 (A) and CA16 (B) in individuals in Guangdong, China, before and after the 2010 HFMD epidemic. Before: before the 2010 HFMD epidemic; after: after the 2010 HFMD epidemic.

The EV71 seroprevalence in different age groups showed an increased trend before 2010 epidemic, except for a dip at 2 years group (Fig. 2A). Compared to those 1 to 3 years old, the proportion of EV71 positive individual was much higher in the 4 years or older groups (P = 0.000011). However, no similar trend of EV71 seroprevalence was observed after the epidemic. The seroprevalence among each age group was not statistically different, although values were slightly increased with age. Compared to the positive rate before the epidemic, the EV71 seroprevalence after the epidemic was much higher in 1 to 3 years groups, especially in 2 years group (P<0.05). Values in other groups did not greatly change.

The age dependent seroprevalence of CA16 before the 2010 epidemic was similar to that of EV71 (Fig. 2B). Except for the reduction at 2 year group, the CA16 positive rate steadily increased from 46.7% at 1 year group to 88% among 5–20 year old. And then it attained its peak. Compared to values before epidemic, the seroprevalence of CA16 after epidemic was not significant changed; only slightly rise or decline was observed in each age group.

### Geometric Mean Titer Distribution of EV71 or CA16 Neutralizing Antibodies

**Figure 3:**
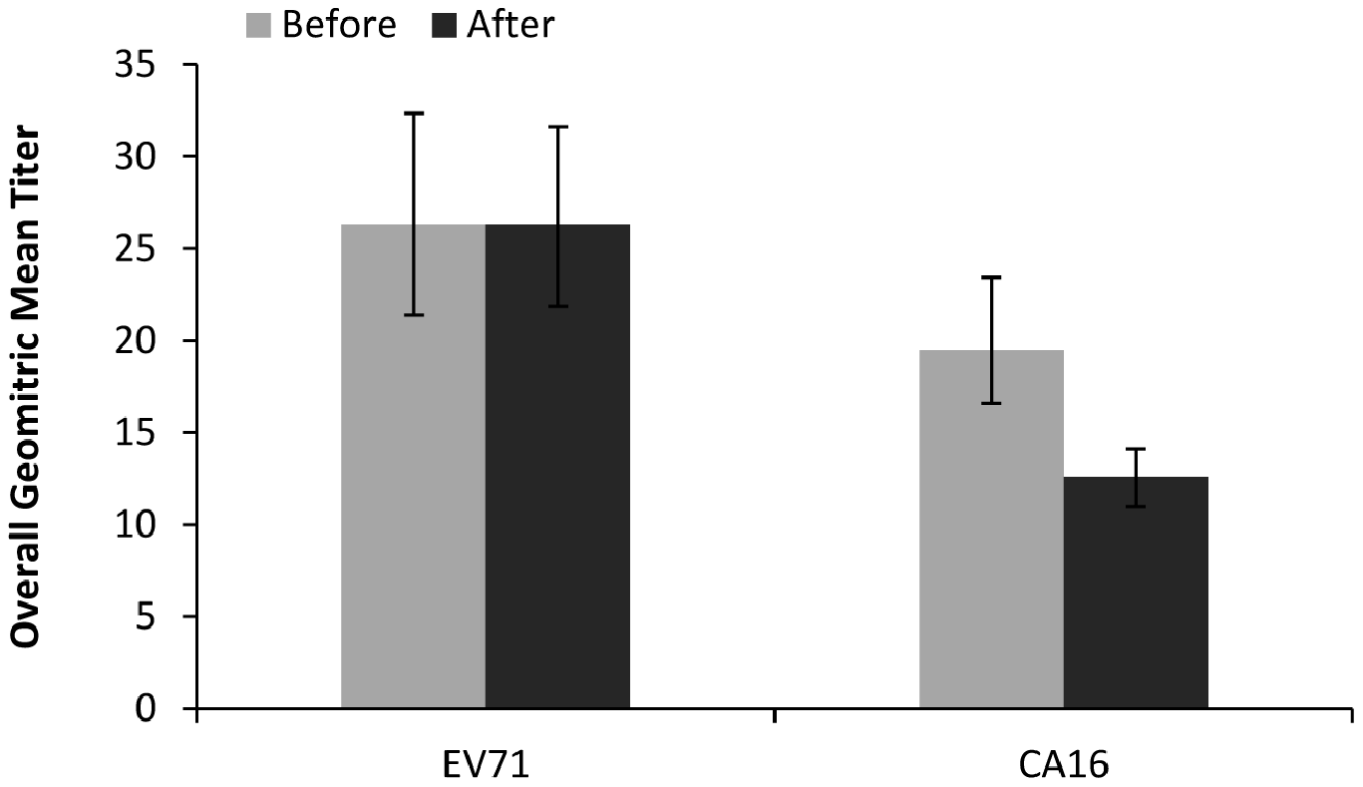
Overall geometric mean titer (GMT) of EV71 and CA16 antibody. Before: before the 2010 HFMD epidemic; after: after the 2010 HFMD epidemic. The lines indicate 95% confidence interval.

**Figure 4:**
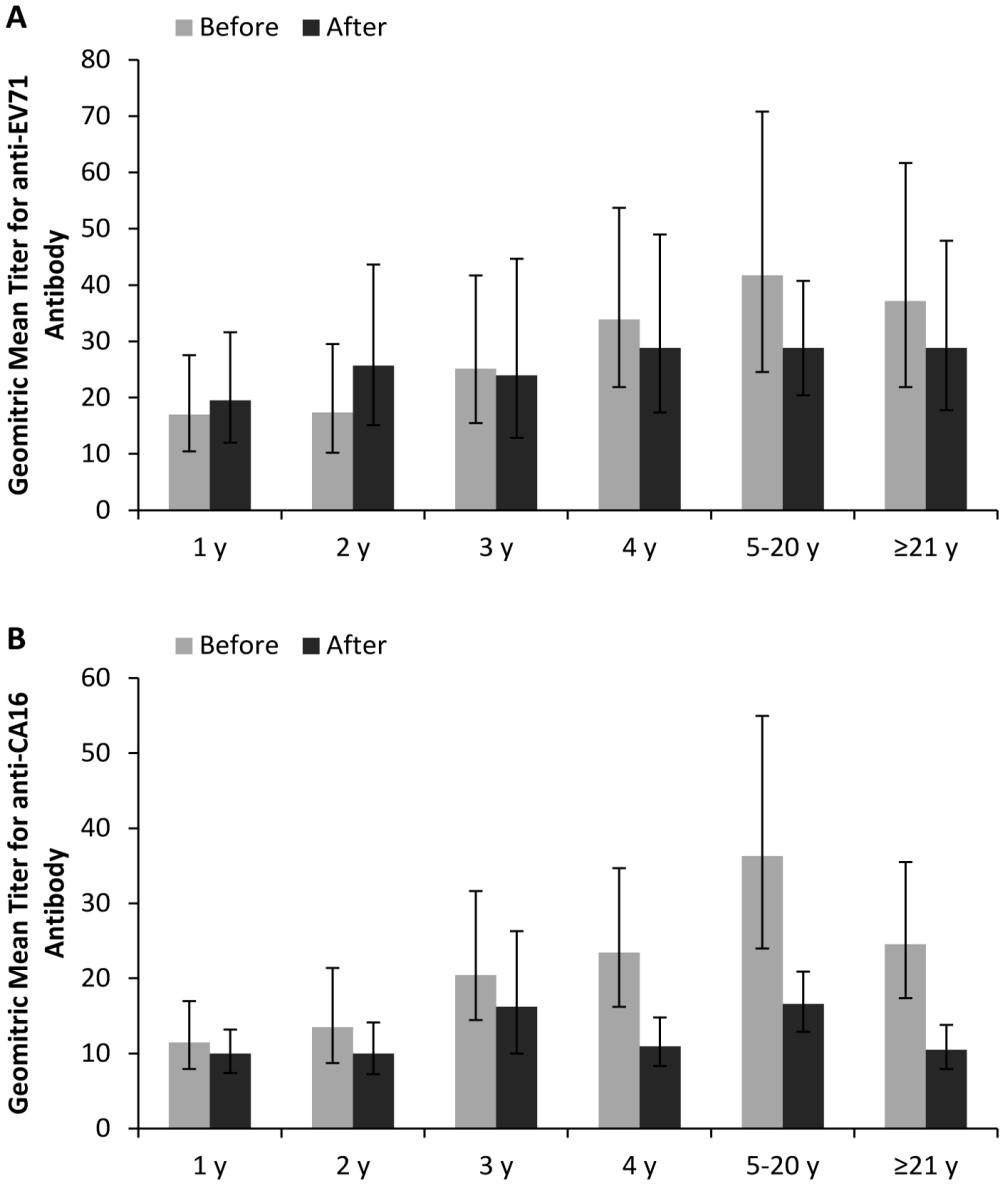
Geometric mean titer (GMT) of EV71 and CA16 antibody by age group. Age-related geometric mean titer (GMT) of EV71 (A) and CA16 (B) antibody in seropositive individuals in Guangdong, China, before and after the 2010 HFMD epidemic. Before: before the 2010 HFMD epidemic; after: after the 2010 HFMD epidemic. The lines indicate 95% confidence interval.

To analyze the immunity level, geometric mean titer (GMT) for EV71 and CA16 neutralizing antibodies was tested. The overall EV71 GMT before and after the 2010 epidemic was almost the same (β = −0.0063, P = 0.92), both had a value of 26.3 (Fig. 3). No significant age related EV71 GMT differences identified, although immune levels for those 1 year old were slightly low (Fig. 4A). While for CA16, the overall GMT was significantly reduced (β = −0.197429, P = 0.000044), with values of 19.5 and 12.6 before and after 2010 epidemic respectively (Fig. 3). Comparisons within each age group showed obviously declined CA16 GMT in individuals aged 4 years or older (P<0.05) after the epidemic (Fig. 4B).

## Discussion

The reasons why frequent HFMD outbreaks occurred in the Asia-Pacific region in the past decade remain unclear. Previous studies suggested that the humoral immunity with neutralizing antibodies is crucial in protecting against EV71 and CA16 infections [10], [31]. Seroepidemiology provides information of great importance to assure population immunity. However, there are still only a few studies have investigated the seroprevalence of EV71 and CA16 in China. In this study, we for the first time described and compared seroprevalence of EV71 and CA16 neutralizing antibodies in Guangdong province, China, in pre- and post- large epidemic of HFMD in 2010.

Our results showed that the overall seroprevalence of EV71 NA was moderate in Guangdong province; about half of the populations had no detectable antibodies against EV71 before the 2010 epidemic. The proportion of positive individuals identified in our results was slightly higher than the values observed in other cities in mainland China, including Lu’an, Shanghai and Jiangsu [19], [24], [25]. The difference might be related to the geographical difference in samples tested. We noticed that the cities included in other published studies were all localized in central China, where shorter summer and relatively low humidity were observed when compared to Guangdong, a subtropical region in the south of China. Considering the associations between EV71 infection and temperature and humidity, the high seroprevalence of EV71 NA in Guangdong may reflect common EV71 infections in this region before 2010 epidemic. Another explanation for the variable results might be related to the age compositions in different tests. The individuals included in other research groups in China were mainly focused on children, while our research tested samples from individuals who were 7 months to 74 years of age. The seroepidemiology of EV71 NA in our study was not limited to children, but reflected the immunological response of general population. The seroprevalence of CA16 NA was similar to that of EV71 NA in Guangdong before 2010 epidemic. This is contrast to other studies showing higher levels of EV71 infections in Guangdong before large-scale outbreaks in 2008 [32]. The high CA16 seroprevalence in our study indicate that frequent CA16 infections without symptoms have occurred in the past. These seropositive individuals that had asymptomatic or unrecognized EV71/CA16 infection may serve as a reservoir for continued viral spread, and should be taken into account when the government develops and implements public health interventions.

Our study also revealed age related increases (except for the 2 years age group) of seroprevalence rates for both EV71 and CA16 before the 2010 epidemic. The seroprevalence rates of EV71 and CoxA16 were significantly lower in children aged 1 to 3 years than in those 4 years or older, which were consistent with previous sero- epidemiological studies in other countries [27], [33]. In Germany, The seroprevalence of the individuals in the 0–3 year and 3–6 year age groups were 27.3% and 45.6%, respectively, with obvious gap observed [34]. A cross-sectional study in Singapore also identified that the seropositive rate increased at an average of 12% per year in pre-school years [28], [35]. The lack of protective antibodies indicated common susceptibility to EV71 and CA16 infections for these age groups. In addition, we also observed that both the age related seroprevalence for EV71 and CA16 NA showed a dip at 2 year age group. The reasons for this lowest positive rate may be as follows: 1) the maternal antibodies which could protect young infants from infectious diseases have declined to undetectable levels for those 2 years old; 2) most children in China are only children and often stay indoors before they go to kindergartens (the usual age is 3–5 years old), thus the chance to acquire infection is low.

Compared to seroprevalence before 2010 epidemic, the EV71 NA seropositive rate after the 2010 epidemic increased significantly. The most obvious increases were observed for individual aged l–3 years, especially for those 2 years old; no significant difference was identified for CA16 NA. As there were no published data about the epidemiology of 2010 HFMD epidemic in Guangdong available, the exact affected individuals and main etiology of the 2010 epidemic in Guangdong remain to be determined by future investigations. Previous studies indicated that immunity level increases when natural infections occur [23], [36]. The seroepidemiological changes identified in this study suggest that EV71 may be the major cause of 2010 epidemic, with the majority EV71 infections in 2010 occurring in 1–3 year age group. Further analysis showed that only male EV71 seroprevalence was significant increased after 2010 HFMD epidemic (55.0% vs. 68.4%). This was highly consistent with previous epidemiology studies in Guangdong. The study showed that boys were more susceptible to enterovirus than girls [20], since they may have more physical activities and be more favoring the spread of HFMD.

The EV71 geometric mean titer (GMT) in different age groups was similar and no significant changes were identified after the 2010 epidemic. This was in contrast to previous studies which found decreased EV71 GMT with age [25], [37]. The steady state of EV71 immune level may attribute to lasting EV71 circulation in mainland China since the 2008. While for CA16, Significantly reduced GMT was identified after the 2010 epidemic, especially in age groups of 4 year and older. Considering the stable proportion of CA16 seropositive individuals after the 2010 epidemic, the lower GMT suggests uncommon CA16 infections during 2010 epidemic, and re-infection of the elderly is rare.

Some limitations are deserved to mention: 1) Study period. According to our previous epidemiology studies, we selected two time periods from January 8 to January 24 and from August 23 to September 4 to represent time periods before and after HFMD epidemic respectively. More time points over a longer time span is needed to identify the seroepidemiological changes before, during and after a HFMD epidemic. 2) Samples composition. All of the serum samples used in this study was stored samples collected for other study (influenza virus study, 2010). Participants’ distribution (age, sex and so on) before epidemic did not well match those after epidemic. If we could perform a perspective study, collect representative samples, we may have obtained more precise conclusions. 3) Lack of etiological information. It would be a good idea that if we could combine seroepidemiology results in our study with etiological information for 2010 HFMD epidemic in Guangdong together. Future studies like this could give a better understanding of the population immunity level.

In conclusion, our results indicate a continuous circulation of EV71 and CA16 in Guangdong before 2010 HFMD epidemic. Only EV71 NA seroprevalence was significantly increased, which suggested a major causative role for EV71 during the 2010 HFMD epidemic. Children less than 3 years old especially those aged 2 years are most susceptible for viral infection and probably are the majority of affected individuals; since they showed the lowest positive rates for EV71 and CA16 NA before epidemic and the most significantly increased EV71 seroprevalence after epidemic. In addition, we noticed lower CA16 GMT values after the 2010 epidemic. Combined with the unchanged seroprevalence for CA16 NA, these results suggest CA16 infections were uncommon in Guangdong during 2010 epidemic.

## Ethical Approval

The whole study was approved by the ethics committee of the Guangdong Provincial Center for Disease Control and Prevention.
